# Macrophage‐derived TNF‐enriched tumour microenvironment shapes pancreatic ductal adenocarcinoma into the basal‐like molecular phenotype through upregulating TAp63

**DOI:** 10.1002/ctm2.1520

**Published:** 2023-12-26

**Authors:** Su Bin Lim, Jae‐Il Choi, Yunjin Go, Yu‐Jin Ha, Min Jae Yang, So‐Hyun Park, Seokhwi Kim, You‐Sun Kim, Dakeun Lee

**Affiliations:** ^1^ Department of Biochemistry and Molecular Biology Ajou University School of Medicine Suwon Republic of Korea; ^2^ Department of Biomedical Sciences Ajou University Graduate School of Medicine Suwon Republic of Korea; ^3^ Department of Pathology Ajou University School of Medicine Suwon Republic of Korea; ^4^ Department of Gastroenterology Ajou University School of Medicine Suwon Republic of Korea

To the Editor:

The molecular classification of pancreatic ductal adenocarcinoma (PDAC) has revealed two subtypes: ‘classical’ and ‘basal‐like’, the latter being linked to squamous histology (high ΔNp63 expression), therapy resistance and a poorer prognosis.^1^, ^2^ However, at the pathologic level, the majority of basal‐like tumours are PDAC‐NOS (not otherwise specified), while adenosquamous carcinoma (ASC) constitutes only a small fraction. Nonetheless, the unique transcriptional signature of ASC, even though they are just a few, hinders the straightforward molecular characterisation of basal‐like PDAC‐NOS. Thus, the key factors responsible for establishing subtype specificity and how these programs are deregulated in the basal‐like PDAC‐NOS remain largely unknown.

To discover the key factors determining the subtype identity in PDAC‐NOS, we used a TCGA‐PAAD dataset previously classified by PurIST^3^ and selected PDAC‐NOS (Figure 1A), which closely aligned with the Moffitt classification (Figure 1B). Among genes highly expressed in the basal‐like PDAC‐NOS (Figure 1C and Figure S1), TP63 appeared atypical as its major isoform, ΔNp63, is specifically associated with squamous differentiation.^4^ Contrary to the increased ΔNp63 expression in transcriptome data (Figure 1D), our immunohistochemistry (IHC) study revealed that ΔNp63 was limited to ASCs, but was not detected in any of the 59 cases of PDAC‐NOS (Figure 1E). Intriguingly, peritumoral normal ducts occasionally expressed ΔNp63, which could be reflected in the transcriptome data. Conversely, we observed that the other TP63 isoform, TAp63, exhibited high expression in the basal‐like tumours (Figure 1F), and its protein expression was confirmed in tissue (Figure 1G and Figure S2). Based on the range of the positive cell proportion, we categorised the cases into TAp63^high^ and TAp63^low^ (Figure 1H). TAp63^high^ PDAC‐NOS was associated with frequent GATA6‐loss and CK5‐positivity, thus representing the basal‐like subtype (Figure 1I,J), which was validated in an external cohort (Figure S3). In the clinicopathologic analyses (Figure S4A–E), TAp63^high^ was associated with poorly differentiated tumours (Figure 1K), higher P53 and PD‐L1 expressions (Figure 1L,M and Figure S4F,G) and a worse prognosis (Figure 1N), as previously indicated.^1^, ^5^, ^6^, ^7^

**FIGURE 1:**
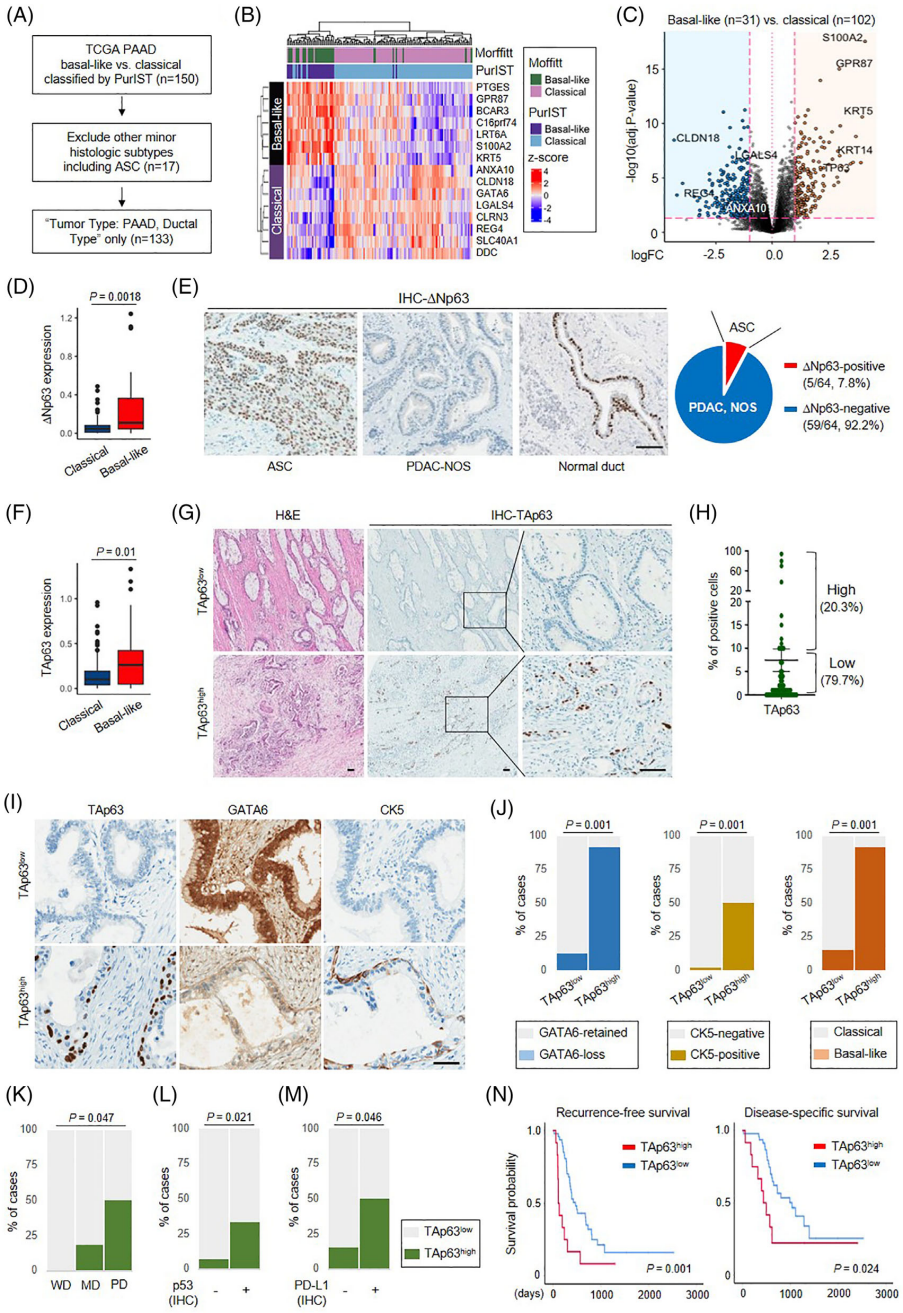
TAp63 overexpression represents the clinicopathologic characteristics of the basal‐like PDAC‐NOS. (A) The overview of the case selection process of TCGA‐PAAD dataset. (B) Heatmap showing different transcriptional profiles between classical and basal‐like subtypes of PDAC‐NOS. (C) Volcano plot demonstrating high expression of basal‐like markers such as S100A2, KRT5 and TP63 in the basal‐like type of PDAC‐NOS. (D) Higher ΔNp63 expression in the basal‐like subtype of PDAC‐NOS of TCGA. (E) Immunohistochemistry study revealed that ΔNp63 expression is only limited to adenosquamous carcinomas (ASCs). PDAC‐NOS did not express ΔNp63 at all. Unexpectedly, we found occasional ΔNp63 expression in normal ductal epithelium adjacent to the tumour. Scale bar: 100 μm. (F) Higher TAp63 expression in the basal‐like subtype of PDAC‐NOS of TCGA. (G) Variable TAp63 expression in a subset of PDAC‐NOS. Scale bar: 100 μm. (H) The proportion of TAp63‐positive cancer cells. Based on this, 20.3% of PDAC‐NOS (≥10% of TAp63) was included in TAp63^high^. Lines indicate mean ± SEM. (I and J) Representative GATA6 and CK5 expression in TAp63^low^ and TAp63^high^ tumours (I) and their correlation analyses (J). Scale bar: 50 μm. (K–M) The correlation analyses between TAp63^high^ and tumour differentiation (K), p53 positivity (L), and PD‐L1 positivity (M). WD: well‐differentiated; MD: moderately differentiated; PD: poorly differentiated. P53 and PD‐L1 were determined by immunohistochemistry in PDAC‐NOS tissues. (N) TAp63^high^ shows significantly worse recurrence‐free survival (RFS) and disease‐specific survival (DSS).

Consistent with previously known characteristics (Figure 2A),^6^ TP63 isoform analysis using PDAC‐NOS of TCGA revealed the enrichment of TGF‐β signalling, Wnt/β‐catenin signalling, glycolysis, hypoxia and inflammatory response, in the TAp63^high^ group (Figure 2B–E). For validation, we transfected TAp63 into pancreatic cancer cell lines and performed RNA sequencing (Figure 2F), which revealed the enrichment of TGF‐β signalling and hypoxia (Figure 2G,H). Furthermore, overexpression of TAp63 alone may be sufficient to induce the basal‐like phenotype in cancer cells (Figure 2I,J). ATAC‐seq analysis revealed similar results (Figure S5). Collectively, these data indicate that TAp63 may play a crucial role in establishing the basal‐like cell identity and contribute to the development of functional characteristics in the basal‐like tumour.

**FIGURE 2:**
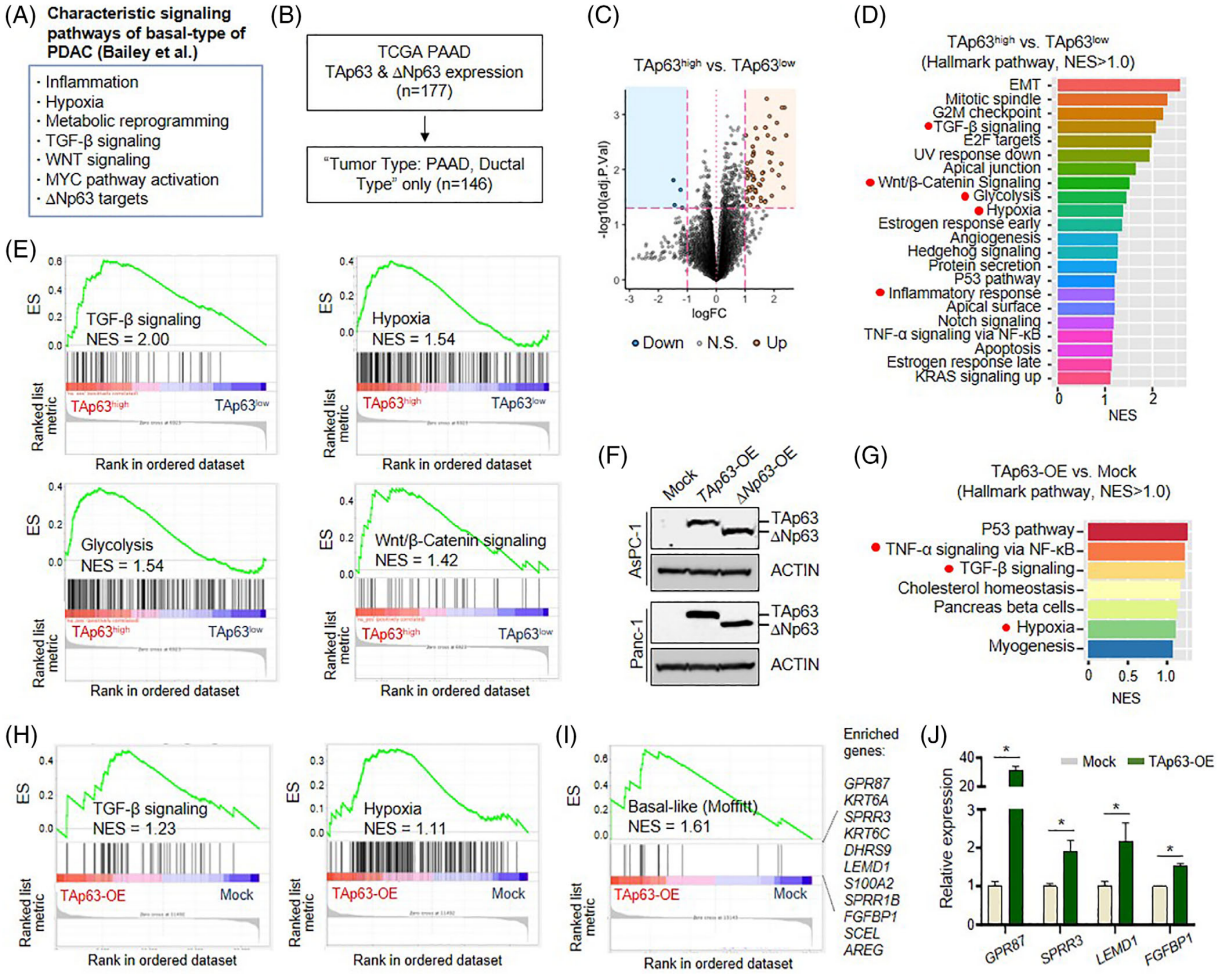
TAp63 is a crucial factor in determining the basal‐like cell state. (A) Summary of the characteristic signalling pathways enriched in the basal‐like subtype by Bailey et al. (B) The overview of the case selection process of TCGA‐PAAD dataset for TP63 isoform analyses. (C) Different gene expression patterns according to TAp63 expression status by volcano plot. (D) Top enriched Hallmark gene sets in TAp63^high^ tumours (25% cutoff). (E) Gene set enrichment analysis (GSEA) shows that TGF‐β signalling, Wnt/β‐catenin signalling, glycolysis, hypoxia, inflammatory response and TNF‐α signalling via NF‐κB (characteristics of the basal‐like subtype) are significantly enriched in TAp63^high^ tumours. (F) TAp63 or ΔNp63 overexpression in cancer cells, confirmed by Western blot. (G) Top enriched Hallmark gene sets in TAp63‐overexpressed cells. (H) Significant enrichment of TNF‐α signalling, TGF‐β signalling and hypoxia in TAp63‐overexpressed cells. (I) Basal‐like gene signature is enriched in TAp63‐overexpressed cells. (J) Validation of selected enriched genes by RT‐qPCR.

Next, we sought the underlying mechanism for aberrant TAp63 expression in cancer cells. To this end, we screened the expression of TAp63 in various types of cancer cells and found that TAp63 expression is quite a rare event in cell lines (Figure 3A and Figure S6A). Additionally, we discovered that TAp63 was expressed in tissue but not in patient‐derived organoid (PDO) (Figure 3B). These findings suggest that TAp63 expression in cancer cells might arise as a consequence of interactions with specific microenvironmental elements.

**FIGURE 3:**
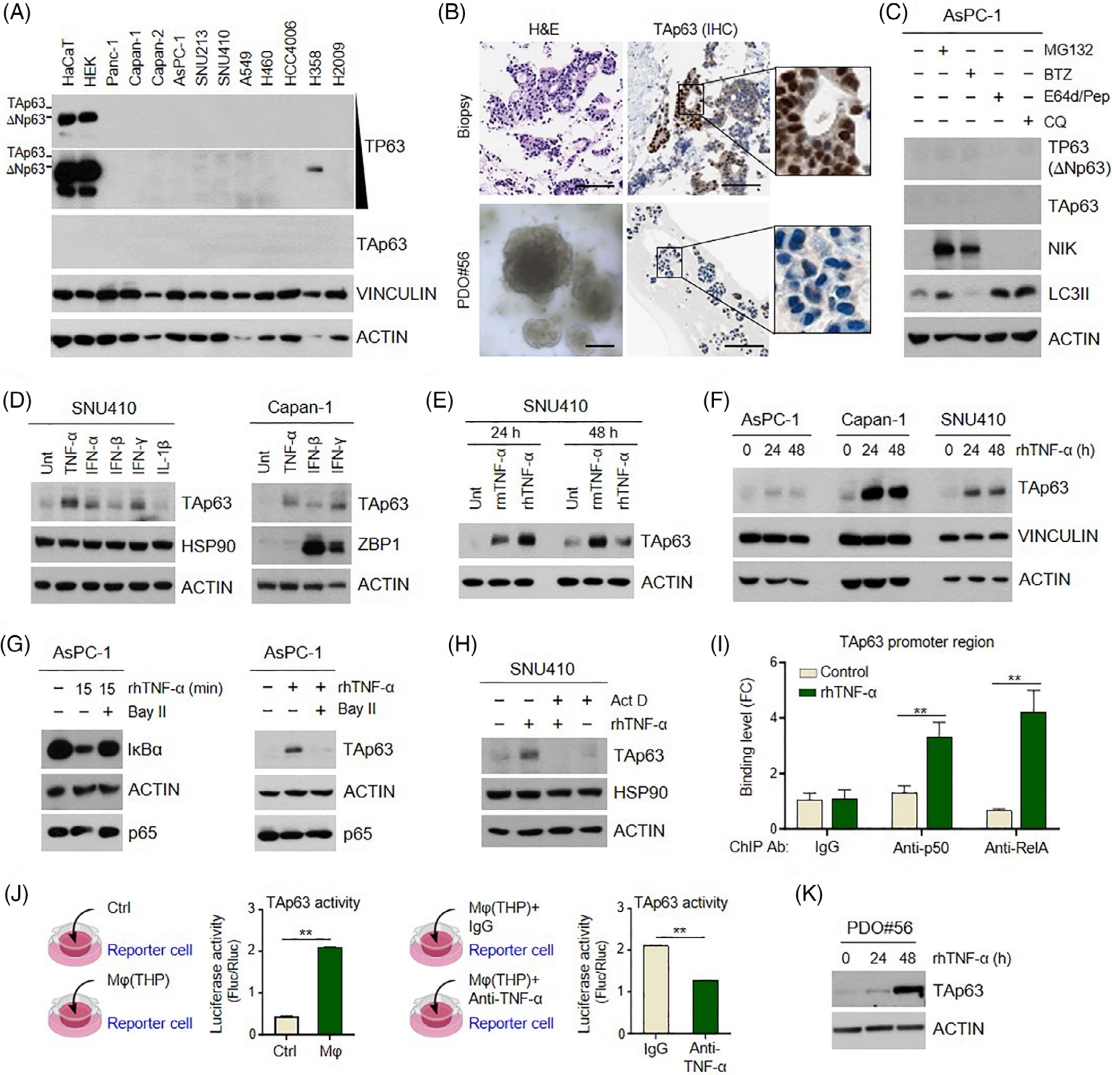
Paracrine TNF‐α upregulates TAp63 expression in cancer cells via NF‐κB. (A) TAp63 expression was explored in various cancer cell lines including PDAC. (B) A case of PDAC‐NOS showed TAp63 expression in tissue, but lost its expression in patient‐derived organoid (PDO#56). Scale bar: 100 μm. (C) ΔNp63 or TAp63 expression was assessed in AsPC‐1 treated with MG132 (10 μM), BTZ (100 nM), E64d/Pep (10 ng/mL), pepstatin A (10 ng/mL) or CQ (50 μM) for 6 h. (D) TAp63 expression was assessed in SNU410 treated with rhTNF‐α (30 ng/mL), rmIFN‐α (200 ng/mL), rhIFN‐β (200 ng/mL), rhIFN‐γ (200 ng/mL) or rhIL‐1β (1 μg/mL) for 24 h. (E) TAp63 expression was assessed in SNU410 treated with rmTNF‐α or rhTNF‐α for 24 or 48 h. (F) TAp63 levels were assessed after TNF‐α treatment in AsPC‐1, Capan‐1 and SNU410. (G) (Left) After 1 h pre‐treatment with Bay II‐7082 (20 μM) to inhibit the NF‐κB pathway, IκBα levels were assessed with or without TNF‐α treatment. (Right) TAp63 levels were assessed after TNF‐α treatment for 24 h in AsPC‐1 pre‐treated with Bay II‐7082 (20 μM). (H) TAp63 levels were assessed after TNF‐α treatment for 24 h in SNU410 pre‐treated with actinomycin D (1 μg/mL) for 1 h to inhibit transcription. (I) After TNF‐α treatment for 6 h in SNU410, chromatin immunoprecipitation (ChIP) followed by qPCR analysis was performed using p50 and RelA antibodies. Two‐way ANOVA with Bonferroni multiple test. (J) (Left) TAp63‐luc reporter cells in the bottom well were indirectly incubated with or without macrophages in the transwell insert for 24 h. Luciferase activity was calculated by the ratio of firefly and Renilla luminescence. (Right) In the indirect co‐culture system, cells were treated with IgG or anti‐TNF‐α for 1 day, then the luciferase activity was measured. Mann–Whitney *U* tests. (K) TAp63 expression was assessed after rhTNF‐α treatment using PDO#56. All data represent mean ± SEM; **p < .05.

Prior studies have suggested that the transcriptional activity of TAp63 can be controlled through the activation of the NF‐κB pathway,^8^ or its expression level may be dependent on the regulation of protein stability.^9^ Considering these factors, we first treated proteasome inhibitors (MG132 and BTZ) or lysosome inhibitors (E64d/Pep A and CQ) on cancer cells, but these did not induce TAp63 expression (Figure 3C). Next, we tested known NF‐κB activators. Among these, TNF‐α showed the most significant increase in TAp63 expression (Figure 3D). Stimulation with either rmTNF‐α or rhTNF‐α also led to the induction of TAp63 protein and RNA expression (Figure 3E and Figure S6B–E) in a time‐dependent manner (Figure 3F). TNF‐α upregulated TAp63 by degrading IκBα, which was reversed by BayII (NF‐κB inhibitor) treatment (Figure 3G and Figure S6F). Moreover, we confirmed that TNF‐α transcriptionally upregulates TAp63 as actinomycin D suppressed this process (Figure 3H).

Next, we conducted Chip and dual‐luciferase assay for validation. In the Chip assay, the direct binding activity of NF‐kB factors (p50 and RelA) on the TAp63 promoter region was increased by treatment with rhTNF‐α (Figure 3I). Subsequently, we explored TAp63 promoter activity using an indirect co‐culture system with macrophages, as macrophages are known to be a major source of TNF‐α in tissue. This assessment revealed a significant increase in TAp63 transcriptional activity induced by the secretome of macrophages, which was reversed upon neutralisation of TNF‐α (Figure 3J). Finally, in PDO#56, we observed the restoration of TAp63 expression upon TNF‐α treatment (Figure 3K). Taken together, these findings indicate that TAp63 expression in cancer cells is induced through the activation of the NF‐κB pathway by paracrine TNF‐α.

Subsequent analysis of single‐cell RNA‐sequencing (scRNA‐seq) data of PDAC^10^ confirmed that cells expressing TNF‐α in cancer tissue were predominantly identified as macrophages, with a gradual increase of TNF‐α in higher clinical stages (Figure 4A). IHC analyses on PDAC‐NOS tissue revealed significantly higher number of myeloid‐derived suppressor cells (MDSCs) and tumour‐associated macrophages (TAMs) in TAp63^high^ tumours (Figure 4B). Consistent with these findings, the spatial distribution of TAp63 expression in PDAC‐NOS tissue was closely associated with TAM infiltration (Figure 4C). Furthermore, the scRNA‐seq data of PDAC showed similar trends (Figure 4D).

**FIGURE 4:**
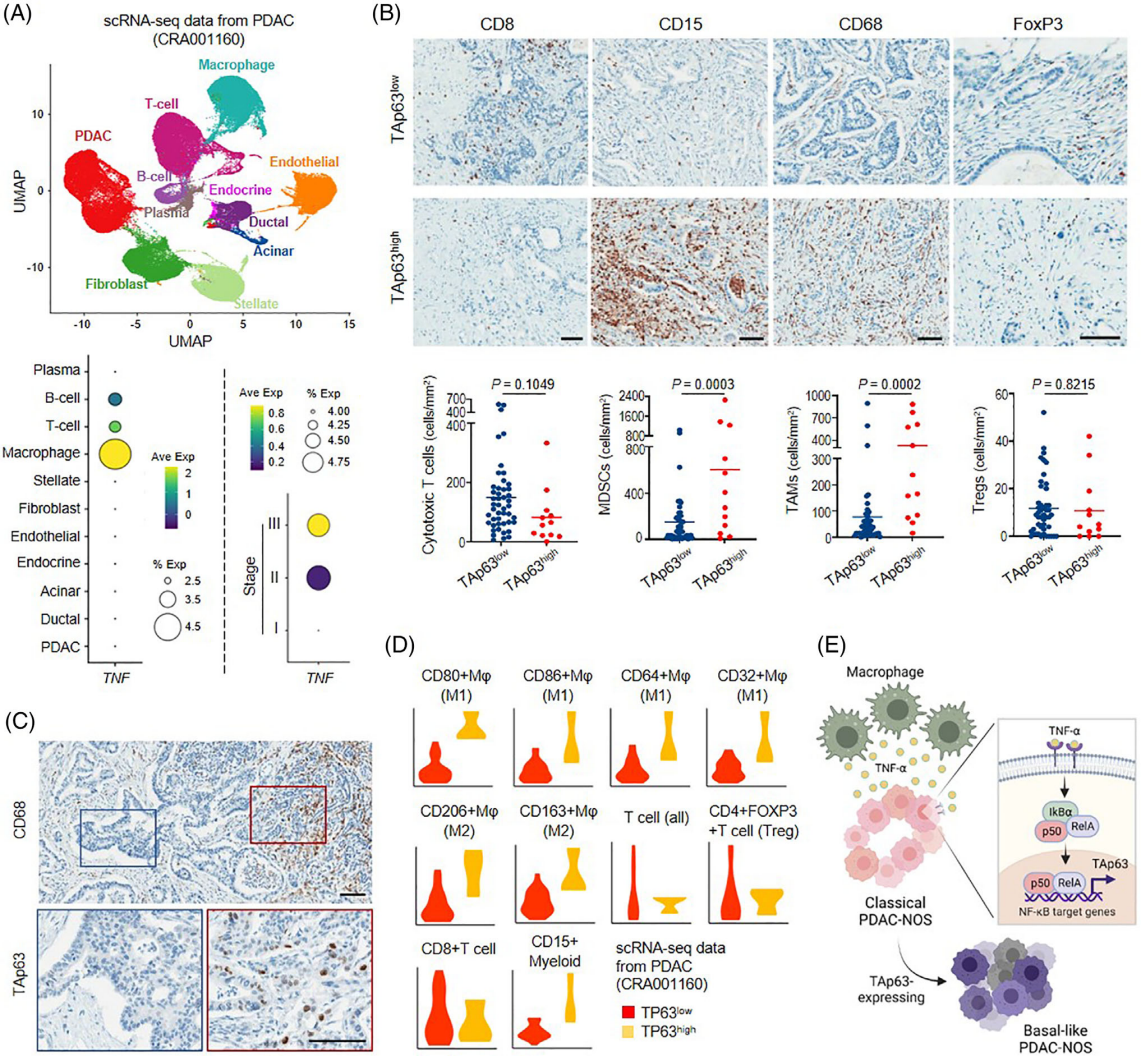
TAp63^high^ tumours are associated with heavy macrophage infiltration in the microenvironment. (A) Single‐cell RNA‐sequencing (scRNA‐seq) analysis using the public dataset (CRA001160). Data confirm that macrophage is a major source of TNF‐α in the tissue. The amount of TNF‐α is correlated with clinical stages. (B) Analyses of immunohistochemistry for CD8 (cytotoxic T cell), CD15 (myeloid‐derived suppressor cell; MDSC), CD68 (tumour‐associated macrophage; TAM) and FoxP3 (regulatory T cell; Treg) according to TAp63 expression status in PDAC‐NOS. Scale bar: 100 μm. (C) Representative image showing that TAp63 expression in cancer cells is correlated with macrophage infiltration. Scale bar: 100 μm. (D) Cell fraction (divided by a total number of cells) of various immune cells in TAp63^low^ (*n* = 21) and TAp63^high^ (*n* = 3) groups using scRNA‐seq data (CRA001160) of PDAC. (E) An illustrative summary of this study. TAp63 is a crucial factor in establishing the basal‐like cell state in PDAC‐NOS, which is induced by TNF‐α‐secreting macrophages in the tumour microenvironment. This image was created with BioRender.com.

Collectively, our study unveils an additional layer of complexity in the basal‐like phenotype of PDAC, highlighting for the first time the crucial role of TAp63 in establishing a basal‐like cell state. Mechanistically, we revealed that the upregulation of TAp63 in cancer cells is triggered by TNF‐α‐induced activation of NF‐κB within the TNF‐α‐secreting TAM‐enriched microenvironment (Figure 4E). These findings provide valuable insights into the fact that the basal‐like phenotype is not solely driven by cell‐intrinsic factors, but is also influenced by cell‐extrinsic inputs. In addition, we propose that future combinatorial treatment strategies targeting the ‘TAM–TNF–basal’ axis should be considered to improve the clinical outcomes for patients with basal‐like tumours in upcoming clinical trials. Ultimately, TAp63 may be used in selecting patients for personalised treatment approaches.

## AUTHOR CONTRIBUTIONS

Conceptualisation: You‐Sun Kim, Dakeun Lee. Data curation: Su Bin Lim, Jae‐Il Choi, Yunjin Go. Formal analysis: Su Bin Lim, Jae‐Il Choi, Yunjin Go, Dakeun Lee. Investigation: Su Bin Lim, Jae‐Il Choi, Yu‐Jin Ha, So‐Hyun Park, Seokhwi Kim, Dakeun Lee. Methodology: Su Bin Lim, Jae‐Il Choi, Min Jae Yang, So‐Hyun Park, You‐Sun Kim, Dakeun Lee. Supervision: Su Bin Lim, Jae‐Il Choi, You‐Sun Kim, Dakeun Lee. Funding acquisition: Su Bin Lim, Jae‐Il Choi, You‐Sun Kim, Dakeun Lee. Validation: Su Bin Lim, Jae‐Il Choi, You‐Sun Kim, Dakeun Lee. Visualisation: Su Bin Lim, Jae‐Il Choi, Yunjin Go, You‐Sun Kim, Dakeun Lee. Writing original draft: Su Bin Lim, Jae‐Il Choi, Dakeun Lee. Writing review and editing: Su Bin Lim, Jae‐Il Choi, You‐Sun Kim, Dakeun Lee. All authors critically reviewed the manuscript, read and approved the final version.

## CONFLICT OF INTEREST STATEMENT

The authors declare that they have no conflicts of interest.

## ETHICS STATEMENT

This retrospective study was approved by the Institutional Review Board of Ajou University Hospital (AJOUIRB‐KS‐2022‐404).

## Supporting information

Supporting Information

Supporting Information

Supporting Information

## Data Availability

Bulk RNA‐seq data that support the findings of this study are deposited in Gene Expression Omnibus (GEO) and can be accessed via GSE218268. Other data are available on reasonable request to the corresponding author.
