# Supplementary material for: Macrophage‐derived TNF‐enriched tumour microenvironment shapes pancreatic ductal adenocarcinoma into the basal‐like molecular phenotype through upregulating TAp63
Source: Clin Transl Med. 2023 Dec 26;13(12):e1520. doi: 10.1002/ctm2.1520 (PMC10751511; doi:10.1002/ctm2.1520)
Supplement: Supplementary file 1 — Supporting Information [file CTM2-13-e1520-s003.docx]

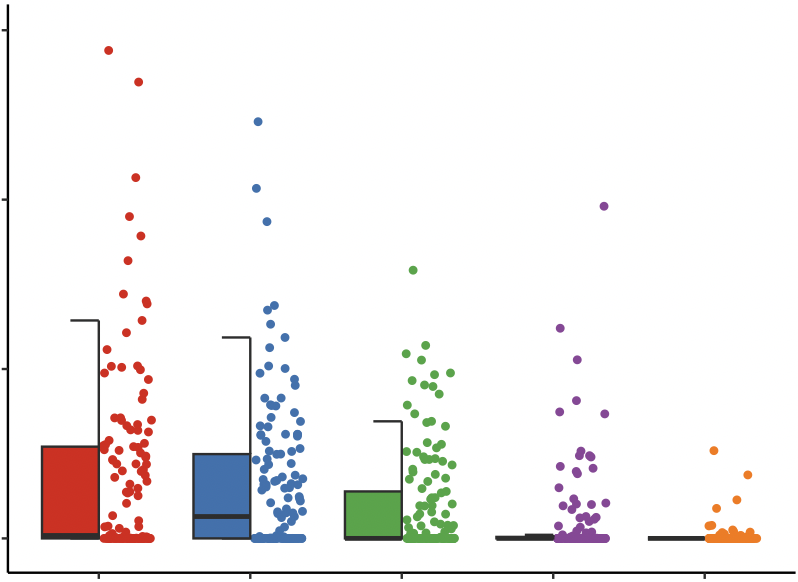


3

2

0

TAp63alpha

log2TPM

TAp63alphaP

TAp63beta

TAp63delta

TAp63gamma

1

isoforms

**Student’s t-test**

TAp63alpha vs TAp63alphaP : p = 0.47

TAp63alpha vs TAp63beta : p = 0.0028

TAp63alpha vs TAp63delta : p = 1.5e-06

TAp63alpha vs TAp63gamma : p = 4.5e-11

TAp63alphaP vs TAp63beta : p = 0.011

TAp63alphaP vs TAp63delta : p = 2.2e-0.6

TAp63alphaP vs TAp63gamma : p = 2.6e-12

TAp63beta vs TAp63delta : p = 0.0074

TAp63beta vs TAp63gamma : p = 2.5e-09

TAp63delta vs TAp63gamma : p = 0.00066

**Supplementary Figure S1.** Analyses for various TP63 isoforms using TCGA-PAAD dataset.


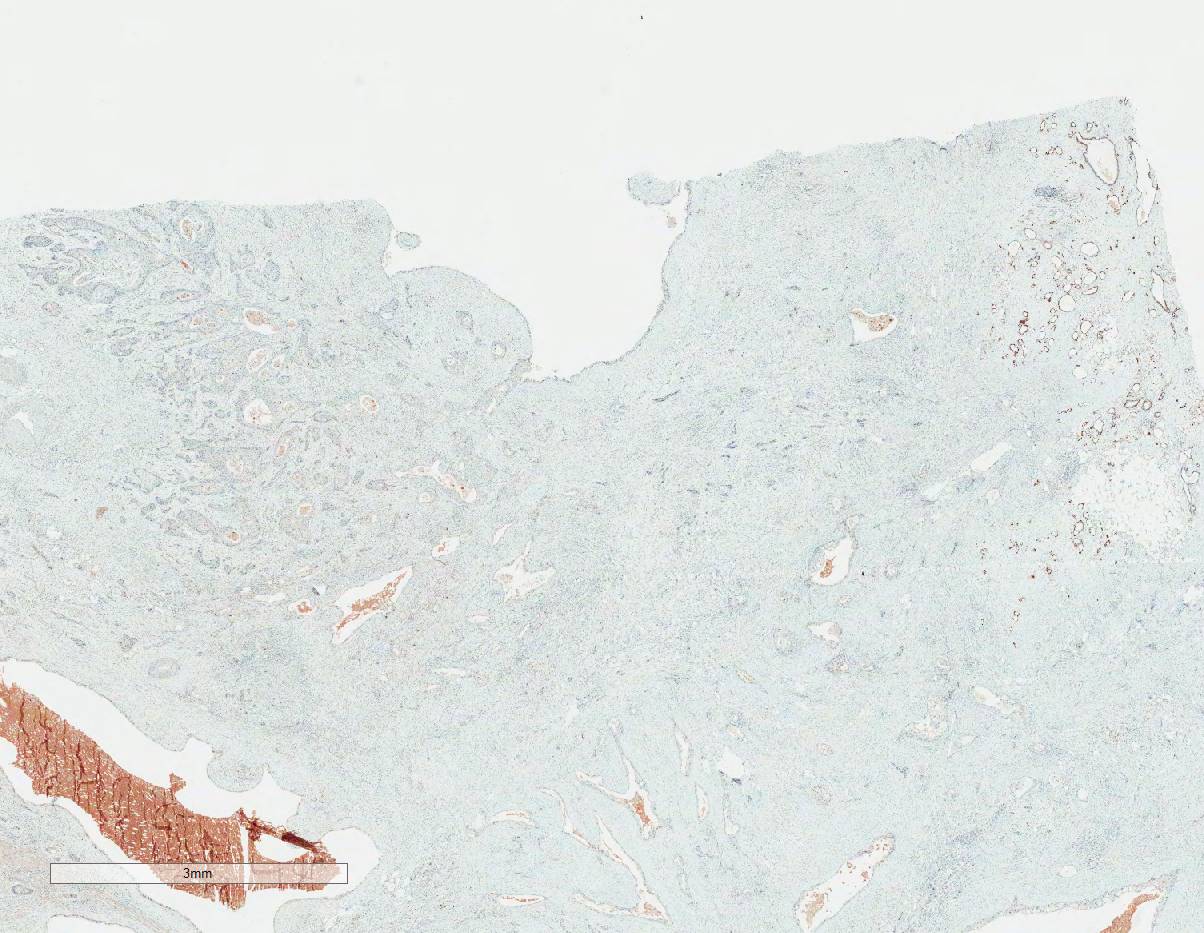

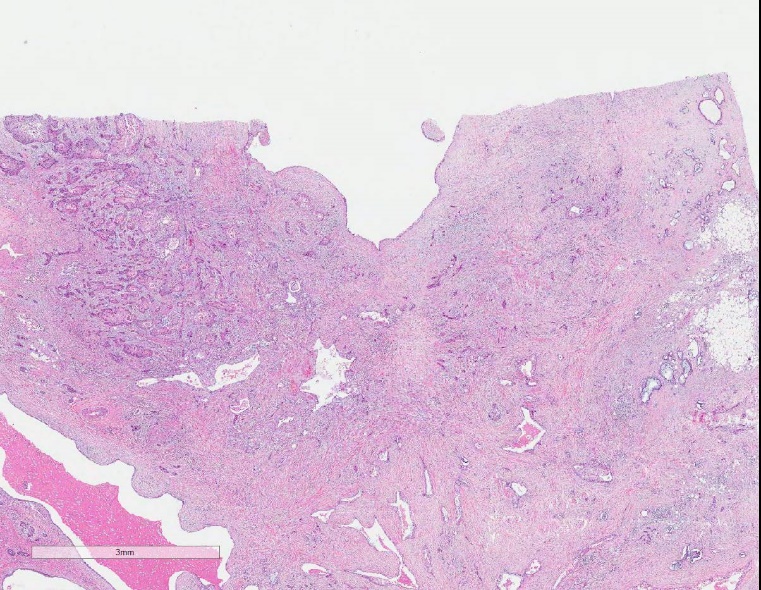

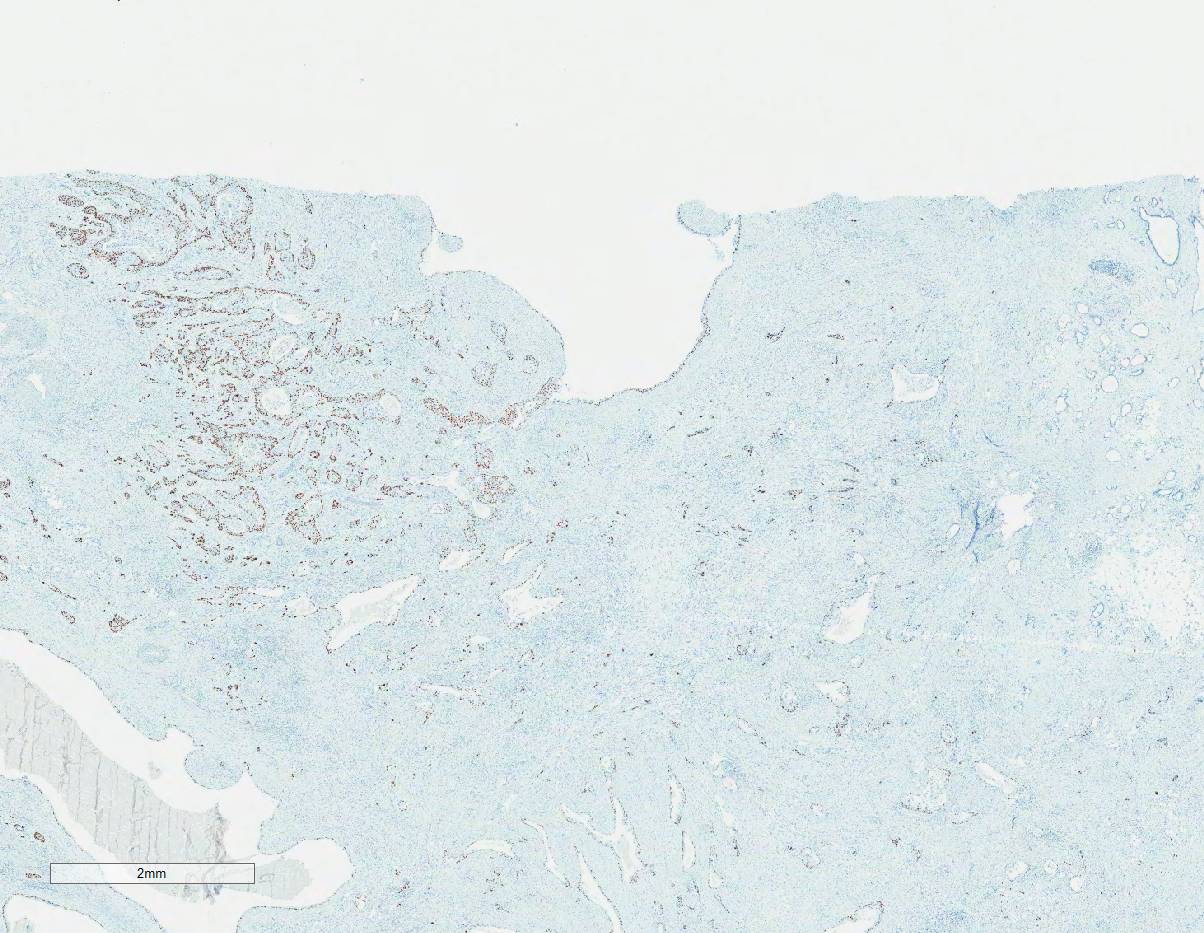

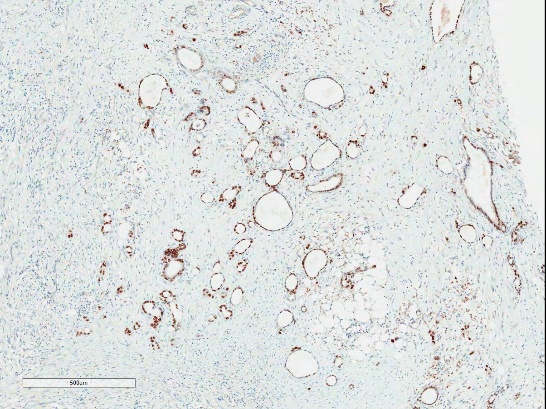

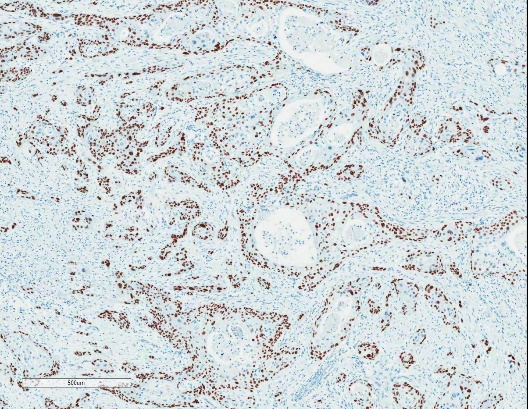

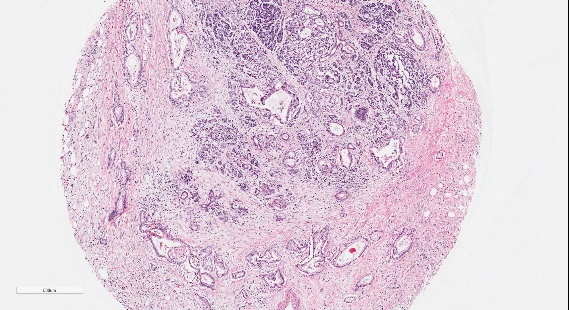

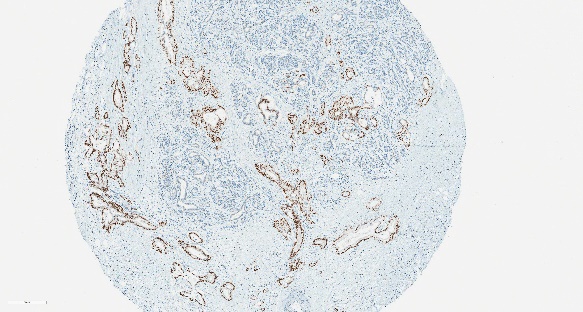

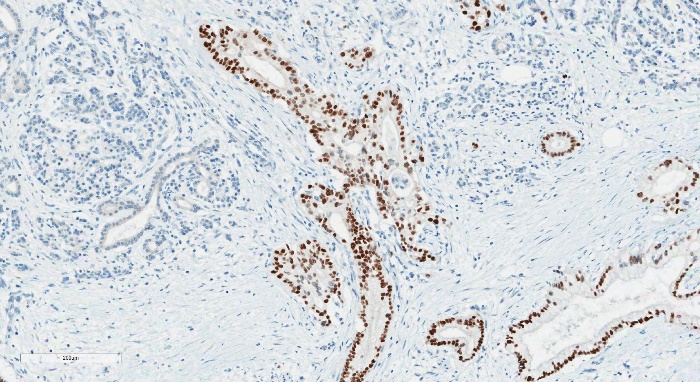

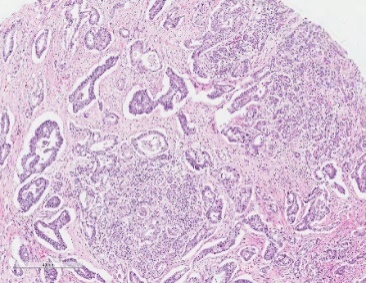

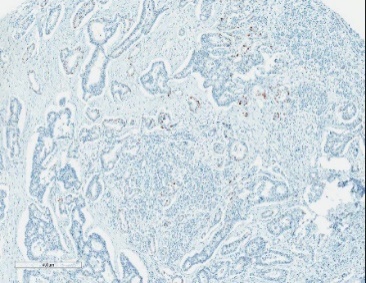

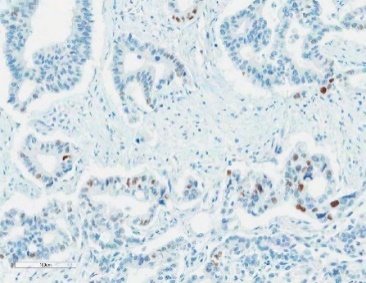


ΔNp63

TAp63

Adenosquamous carcinoma

H&E

ΔNp63

TAp63

‘Squamous’

‘Adenocarcinomatous’

TAp63^low^

IHC-TAp63

H&E

TAp63^high^

**A**

**B**

**Supplementary Figure S2. A.** Representative photomicrographs showing various TAp63 expression in PDAC-NOS. Scale bars: 100 μm. **B.** Representative photomicrograph of a case of adenosquamous carcinoma. Immunohistochemistry study shows ΔNp63 expression in squamous carcinoma region, whereas TAp63 expression was almost exclusively observed in adenocarcinoma area. Scale bars: (left) 1 mm, (right) 100 μm.


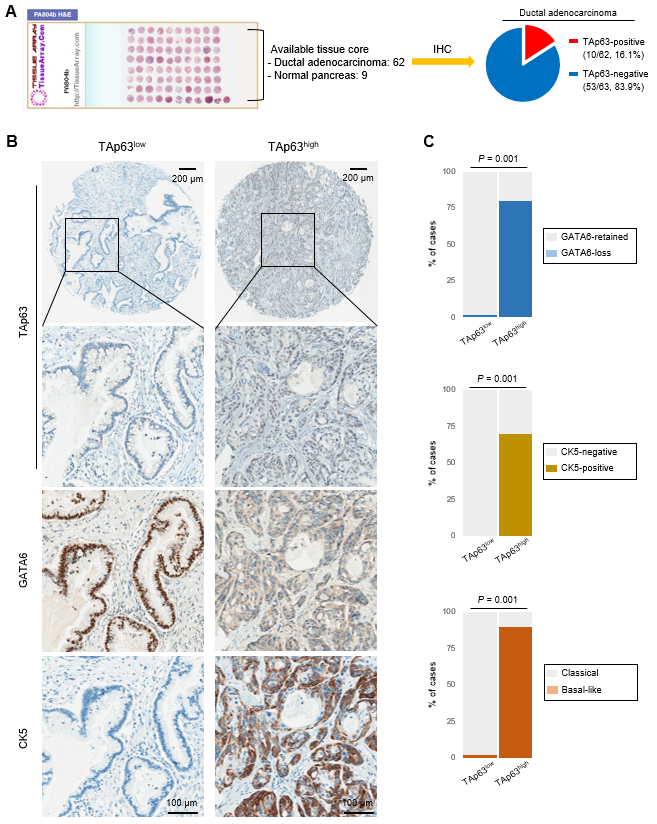


**Supplementary Figure S3. A.** Commercial tissue microarray slides (PA804b, TissueArray.com) containing 71 available tissue cores were used to validate our results. These include 62 cases of pancreatic ductal adenocarcinoma (PDAC-NOS) and 9 normal pancreas tissue. Ten out of 62 PDAC-NOS cases were stained with TAp63. **B.** Representative photomicrographs for TAp63, GATA6, and CK5 expression in PDAC-NOS. **C.** The correlation analyses between TAp63^high^ and GATA6-loss, CK5-positivity, or basal-like phenotype. Cases showing either GATA6-loss or CK5-positivity rendered the basal-like type in this analysis.


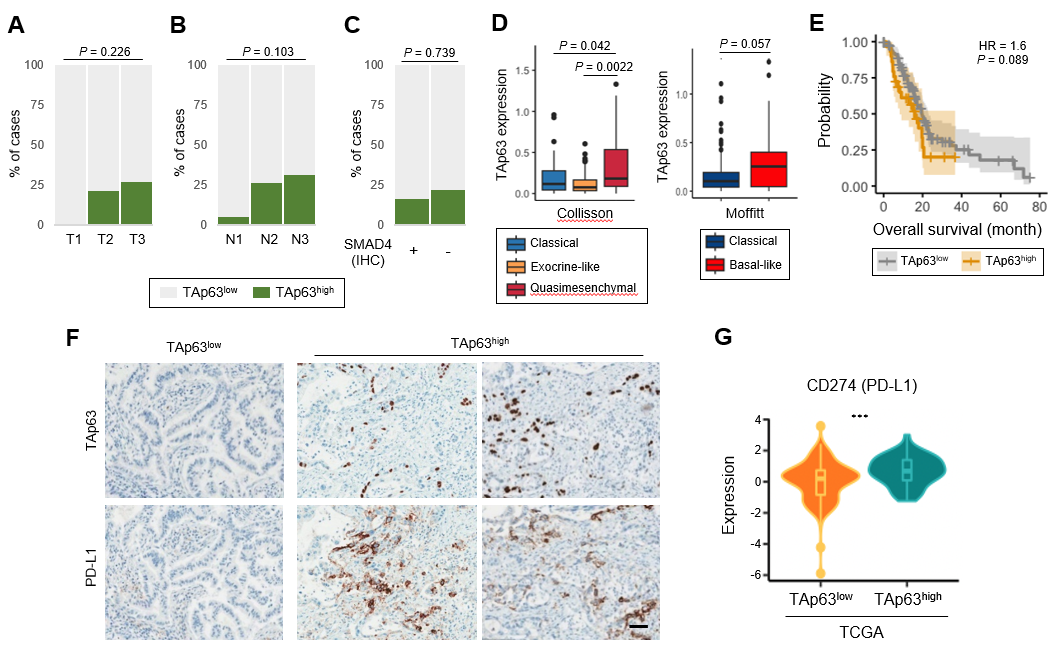


**Supplementary Figure S4.** The correlation analyses between TAp63^high^ and T stage (**A**), N stage (**B**), and SMAD4-loss determined by immunohistochemistry (**C**). **D.** TAp63 expression is also higher in Collison “quasimesenchymal” and Morffitt “basal-like” of PDAC-NOS of TCGA. **E.** TAp63^high^ shows a trend toward worse overall survival in PDAC-NOS of TCGA data. **F.** Representative photomicrograph showing different PD-L1 expression according to the TAp63 status in PDAC-NOS. **G.** CD274 (PD-L1) expression levels in TAp63^low^ and TAp63^high^ tumors in TCGA-PAAD. The cutoff for TAp63^high^ is 25%. ****P* < 0.001.


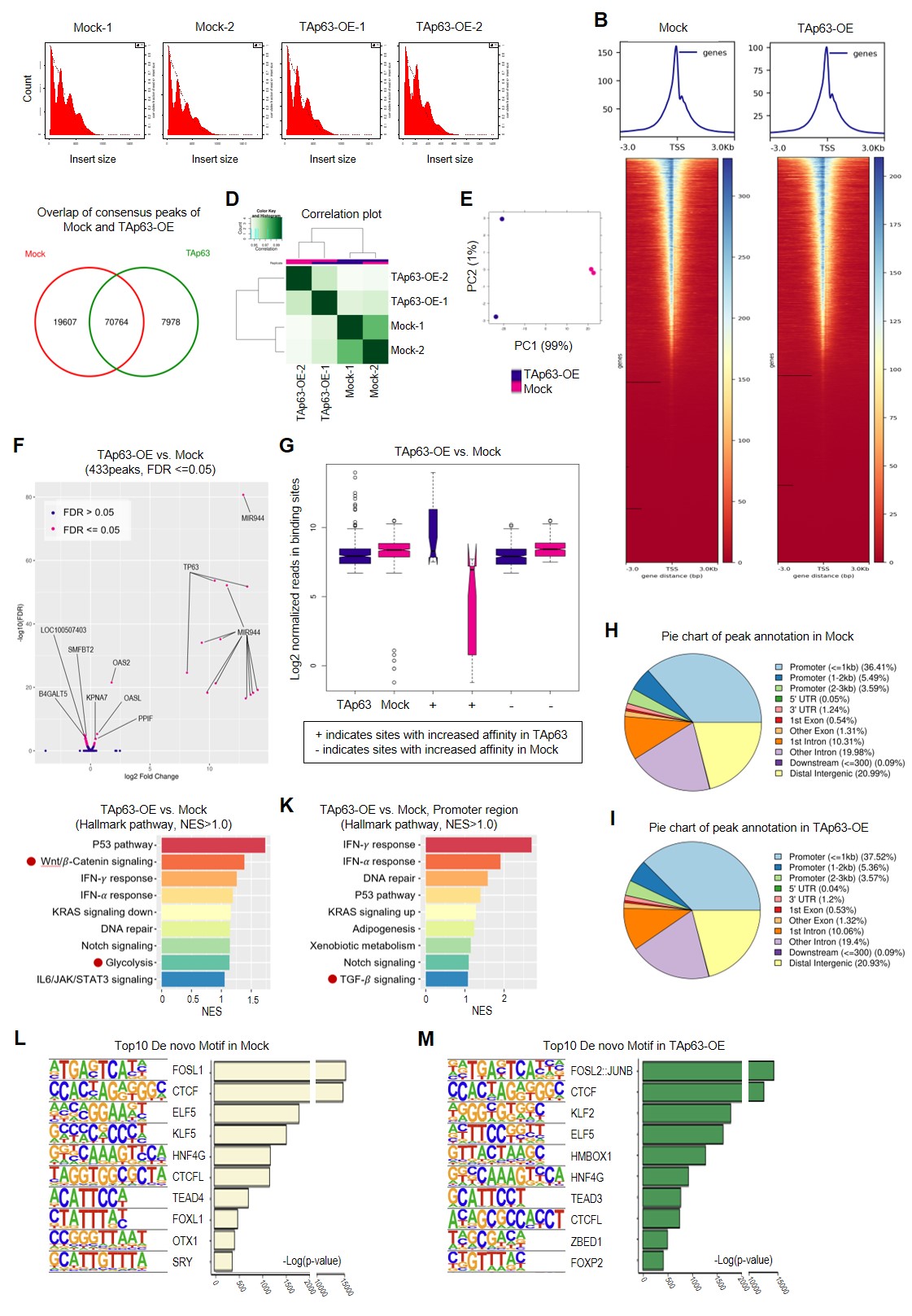


**Supplementary Figure S5.** **A.** The distribution of ATAC-seq fragment lengths in two replicates of both mock and TAp63-OE group reveals the periodic signal of nucleosomes. **B.** Heatmaps depicting accessibility around ATAC-seq peaks. **C.** A Ven diagram reveals 19,607 mock-specific peaks, 7,979 TAp63-OE-specific peaks, and 70,764 consensus peaks through peak calling and differential peak analysis. **D.** The correlation plot demonstrates replicates of TAp63 and mock groups clustering closely together. **E.** Principal component analysis (PCA) demonstrates that the predominant source of variances in the ATAC-seq data was between mock and TAp63-OE groups, with PC1 accounting for 99% and PC2 for 1% of the variation. **F.** Analysis of differential peaks between TAp63-OE and mock groups reveals the increased expression not only in TP63 and MIR944, situated within an intron of TP63 but also in genes such as OAS2, OASL, PPIF, and KPNA7. **G.** A notable increase in normalized reads within binding sites exhibiting enhanced affinity in TAp63-OE samples compared to mock samples. **H-I.** Pie charts depicting the relative location of peaks concerning the nearest annotated gene demonstrate a frequent occurrence of peaks in the promoter region of both mock (**H**) and TAp63-OE groups (**I**). **J-K.** The enriched GSEA hallmarks pathways in the TAp63-OE group compared to the mock group by gene ontology analysis of differentially expressed peaks. The characteristic signaling pathways of the basal-like subtype were highlighted using a red dot. **L-M.** *De novo* motif analyses inferred potential key transcription factors (TFs) associated with mock (**L**) and TAp63-OE groups (**M**), respectively. TFs including FOSL2::JUNB, KLF2, HMBOX1, TEAD3, CTCFL, ZBED1, and FOXP2 were only specifically enriched in the TAp63-OE group.


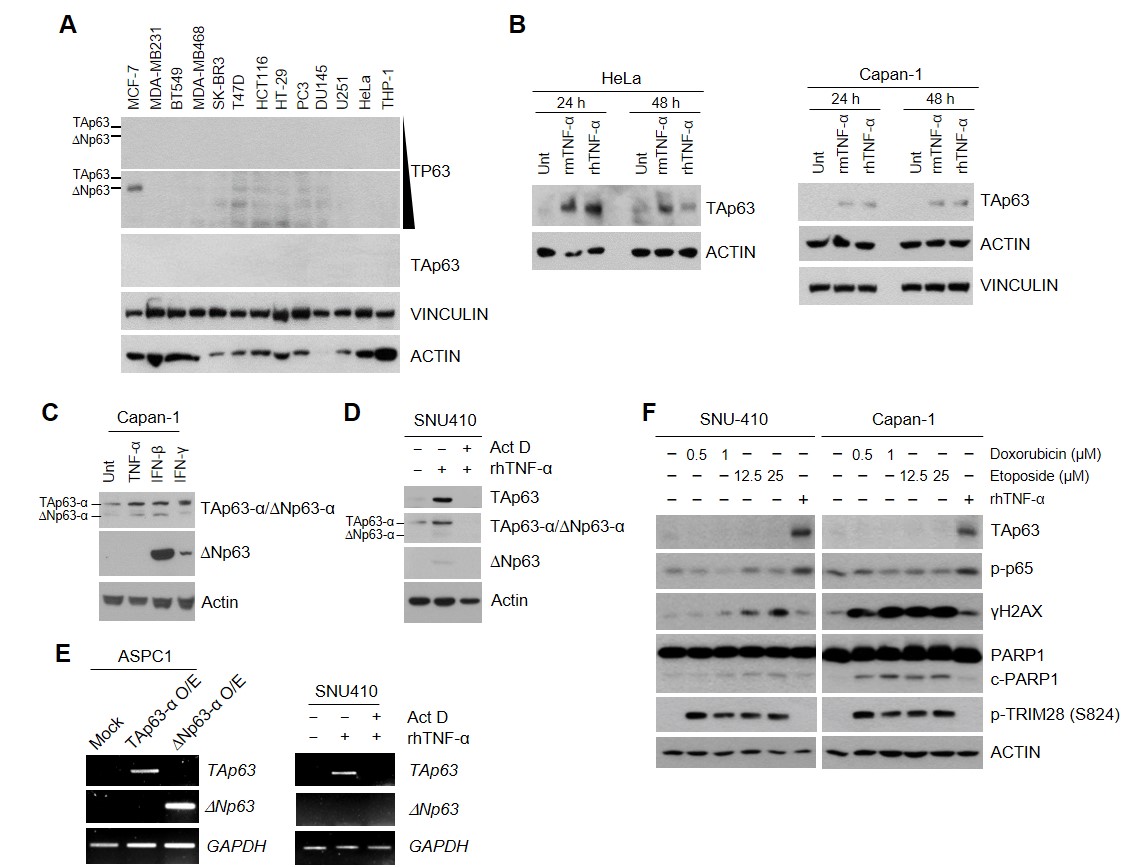


**Supplementary Figure S6.** **A.** Expression of TP63 isoforms including ΔNp63 and TAp63 in various cancer cell lines were assessed using western blot. ACTIN and VINCULIN were used as a loading control. **B.** HeLa and Capan-1 cells were treated with rmTNF-α (30 ng/ml) and rhTNF-α (30 ng/ml) for the indicated time duration, and western blot was performed to detect the TAp63 expression. **C.** Capan-1 cells were treated with rhTNF-α (30 ng/ml), rhIFN-β (200 ng/ml), rhIFN-γ (200 ng/ml), and western blot was performed to recognize TAp63-α and ∆Np63-α. **D.** After pre-treatment with Actinomycin D (1 μg/ml) for 1hr to inhibit transcription, TAp63 isoform levels were analyzed in SNU410 cells following treatment with rhTNF-α. **E.** (Left) Primer design and PCR conditions were validated using TAp63 and ∆Np63 overexpressing cells. (Right) Quantification of TAp63 and ∆Np63 mRNA levels by exposure of rhTNF-α with pre-treated Actinomycin D (1 μg/ml) in SNU410 cells. **F.** TAp63 expression was assessed after treatment of two DNA-damaging agents; Doxorubicin and Etoposide. DNA-damaging response and the transcriptional regulation in response to DNA damage were indicated by γH2AX and p-TRIM28, respectively.
