# Supplementary material for: Macrophage‐derived TNF‐enriched tumour microenvironment shapes pancreatic ductal adenocarcinoma into the basal‐like molecular phenotype through upregulating TAp63
Source: Clin Transl Med. 2023 Dec 26;13(12):e1520. doi: 10.1002/ctm2.1520 (PMC10751511; doi:10.1002/ctm2.1520)
Supplement: Supplementary file 2 — Supporting Information [file CTM2-13-e1520-s002.docx]

**Supplementary Methods**

**The Cancer Genome Atlas (TCGA) analysis**

RNA-seq data and the associated clinical data derived from the TCGA study of PDAC (TCGA-PAAD) were processed as previously described.^1,2^ Briefly, the mRNA expression data (RNA Seq V2 RSEM) and the associated clinical data were downloaded directly from the open platform cBioPortal. The TP63 isoform expression data were provided by the authors of a previously published study.^3^ Kaplan–Meier (KM) survival curves were derived for overall survival (OS) and disease-specific survival (DSS) endpoints using the R survival package (v3.2.11). The computed score represents the mean value of the expression of genes used to define each PDAC subtype. The PurIST classification file was provided in **Supplementary Table S1** in the original paper.^4^

**DE and GSEA analysis**

DE analyses were performed using the linear modeling features of the R limma package (v3.46.0). Statistical cutoffs of |log2FC| > 1 and adjusted *p*-value < 0.05 were applied to determine genes that were differentially expressed in the TP63-high group. Gene set enrichment analysis (GSEA) was performed using the GSEA software (v4.3.2).^5,6^ The pre-ranked gene list (ranked by log2FC) was loaded into the software and the Hallmark pathways (h.all.v2023.1.Hs.symbols.gmt) of the Molecular Signatures Database (MSigDB) were used for all GSEA analyses. The number of permutations was set to 1000.

**Single-cell RNA-sequencing (scRNA-seq) analysis**

Processed scRNA-seq data derived from primary tumor tissues of 24 PDAC patient samples were obtained using the accession code CRA001160.^7^ The expression matrix of QC-passed cells and cell types previously defined by the authors were used as input data in R Seurat (v4.2.0).^8^ For preprocessing, a standard Seurat pipeline was used, including log normalization (scale factor = 10,000), finding variable genes (vst, 2,000 features), data scaling, PCA, finding neighbors (20 PCs), clustering of cells, and visualization of cells by non-linear dimensional reduction (UMAP) with author-defined cell type annotation. Cells from normal pancreatic tissues were excluded from the analysis.

**Patients**

A total of 64 patients with pancreatic cancer (PDAC-NOS, 59 cases; ASC, 5 cases) who underwent diagnostic EUS-guided biopsy and subsequent surgical resection between February 2015 and March 2021 at Ajou University Hospital were enrolled in this study. Patients who had received preoperative chemotherapy and/or radiotherapy were excluded. Pathological stages were determined according to the AJCC 8^th^ edition.

**Immunohistochemistry (IHC)**

IHC was conducted on formalin-fixed, paraffin-embedded, 4 µm-thick tissue sections using a BenchMark XT autoimmunostainer (Ventana Medical Systems, Tucson, AZ, USA). The primary antibodies used in this study were as follows: TAp63 (1:1000; Novusbio, Centennial, CO, USA), ΔNp63 (p40; Roche, Basel, Switzerland), PD-L1 (22C3; DakoCytomation, Glostrup, Denmark), FoxP3 (1:500; Abcam, Cambridge, MA, USA), CD8 (Roche), CD68 (1:2000; DakoCytomation), CD15 (Roche), p53 (Roche), SMAD4 (1:1000; Abcam), GATA6 (1:1000; R&D systems), and CK5 (1:400: Leica). For validation, we purchased commercial tissue microarray slides containing pancreatic cancer and normal pancreas tissue (PA804b, TissueArray.com) and performed IHC on these slides.

**Interpretation of IHC results**

Positivity for TAp63 or ΔNp63 was evaluated on whole slide scanning images, and the positive cell proportion was blindly measured by two expert pathologists (S. Kim and D. Lee). Discordant cases were re-evaluated using the image analysis software QuPath (v0.3.0) on pre-annotated tumor sections. Regarding immune cell counts, quantitative analyses were done on three representative regions (each with an area of 0.5 mm^2^) using QuPath software. Tumors showing membranous staining for PD-L1 in more than 5% of tumor cells with any intensity were considered positive, as previously suggested.^9,10^ p53 was considered positive when tumor cells showed strong nuclear staining in ≥ 50% of tumor cells, while the remainder were considered negative. SMAD4 is normally stained in the cytoplasm with weak-to-moderate intensity, and this was considered “retained.” Complete loss of cytoplasmic staining for SMAD4 was rendered “loss.” Loss of nuclear expression in ≥ 10% of tumor cells was considered “loss” for GATA6, while positive nuclear staining in ≥ 10% of tumor cells was rendered “positive’ for CK5.

**Cell lines**

Human pancreatic cancer cell lines AsPC-1, Capan-1, Capan-2, PANC-1, SUN213, SNU324, and SNU410 were purchased from the Korean Cell Line Bank (KCLB). SK-BR3, MCF-7, MDA-MB231, MDA-MB468, U251, BT549, HCT116, HT-29, HeLa, Raji, A549, H460, HCC4006, H358, H2009, T47D, DU145, and PC3 cells were purchased from American Type Culture Collection (ATCC, Virginia, USA). The Cells were maintained in RPMI 1640 (Welgene Inc., Gyeongsanbuk-do, Korea) or DMEM (Welgene Inc.) supplemented with 10% fetal bovine serum (FBS, Welgene Inc.), 1% penicillin, and streptomycin at 37 °C in a humidified atmosphere containing 5% CO_2_ according to manufacturer’s guideline, appropriately.

**Real-time quantitative RT-PCR**

Total RNA was extracted from cancer cells using the RNeasy Plus Mini Kit (Qiagen, Hilden, Germany) according to the manufacturer’s instructions. RNA quantity and quality were examined using a Synergy H1 microplate reader (BioTek, Winooski, VT, USA), and cDNA was synthesized using AccuPower RT PreMix (BioNeer, South Korea), according to the manufacturer’s instructions. Real-time quantitative RT-PCR (qRT-PCR) was performed using SYBR (Takara, Japan) and a Thermal Cycler Dice Real-Time System III device (Takara). Primer information is provided as below.

| **Primer** | **Sequence** |
| --- | --- |
| ***GPR87*** | Forward 5’-GTTCAACTTGACGCTTGCAAAATTAC-3 |
|  | Reverse 5’-GTTGCCTGAATTGTGACTCTCTTG-3 |
| ***SPRR3*** | Forward 5’-AATATTTGTTCCCACAACCAAGGAG-3 |
|  | Reverse 5’-CTGGAATCTTTGTGTTTCCAGGTT-3 |
| ***LEMD1*** | Forward 5’-GAGTGACTGTAAATTGCAGAACCAA-3 |
|  | Reverse 5’-GAGGTGAGACCAACAACTGTACTAA-3 |
| ***FGFBP1*** | Forward 5’-ATGAATTTTCCTGTGTCTTTGCTGG -3 |
|  | Reverse 5’-ATTCTGGAAAATCCTTTCTGCACAC -3 |
| ***GAPDH*** | Forward 5’-TTGTCAAGCTCATTTCCTGGTATG-3 |
|  | Reverse 5’-TCTCTCTTCCTCTTGTGCTCTTG-3 |

**For semi-quantitative RT-PCR**

PCR reactions from cDNA were performed using EmeraldAmp® PCR Master Mix (Takara Bio, Tokyo, Japan). To determine level of TAp63 and ΔNp63, we designed PCR forward primers which specifically annealed to the exon 3A (TA) or 3B (TA*). The following primers were used: *TAp63* (Forward: CAAGATTGAGATTAGCATGGACTGT, Reverse: GGGTGATGGAGAGAGAGCATC), *ΔNp63* (Forwad: TTGTACCTGGAAAACAATGCCC, Reverse: GGGTGATGGAGAGAGAGCATC). *GAPDH* (Forward: ATGTTCCAATATGATTCCACCCATG, Reverse: TTGTCATACTTCTCATGGTTCACAC).

**TAp63 and ΔNp63 overexpression**

The TAp63 and ΔNp63 coding sequences were gifts from David Sidransky (plasmids #27008 and #26979; Addgene, Watertown, MA, USA).^11^ These sequences were subcloned into the pHTC HaloTag CMV-neo vector (Promega, Madison, WI, USA) using the standard restriction enzyme method. The TAp63, ΔNp63, and mock vectors were transfected into AsPC-1 and Panc-1 cells using Lipofectamine 3000 transfection reagent, according to the manufacturer’s instructions (Thermo Fisher Scientific, Waltham, MA, USA). After 48 h, the transfection efficiency was analyzed using qRT-PCR.

**ATAC-seq Data Processing**

Raw fastq files were assessed for quality, adaptor content, and sequencing quality using FastQC v0.12.1. Adaptor sequences were trimmed using cutadapt 4.5. Paired-end ATAC-seq reads were aligned to the human reference genome (hg38) from UCSC using Bowtie2 v2.5.2. Reads from mitochondrial DNA and the X and Y chromosomes were filtered using samtools v1.18. Properly paired reads with a high mapping quality (MAPQ score > 30) were obtained via deeptools v3.5.4. Duplicate reads were removed using Picard v2.27.5 (http://broadinstitute.github.io/picard/) MarkDuplicates. Reads were shifted +4 base pairs (bp) and -5 bp on the + and - strands with the alignmentSieve of deeptools, respectively, to remove bias from the insertion of adapter sequences by Tn5 transposase. Data quality was confirmed using Picard CollectInsertSizeMetrics for each sample.

**Peak Calling**

Replicate files were merged for each group using samtools. ATAC-Seq peak regions of merged files were called using MACS2 v2.2.9.1 with parameters --nomodel --shift -75 --extsize 150 --call-summits --keep-dup all. Blacklisted regions were excluded from called peaks (https://www.encodeproject.org/annotations/ENCSR636HFF/) using bedtools v2.31.1. Quality of peak files was checked using ComputeMatrix v3.5.4 and plotHeatmap 3.5.4.

**Downstream Analysis for ATAC-seq Data**

Downstream analysis was performed using Diffbind v3.10.1 in R v4.3.1. BAM files and multi-replicate peak files were loaded into a large DBA object using the ‘dba.count’ function of Diffbind. Differences in peak data between TAp63-OE and Mock groups were confirmed with a Venn diagram, correlation plot, and PCA plot. Differential accessibility regions (DAR) were identified via Diffbind ‘dba.analyze’, with the following statistical cutoff (FDR < 0.05).

**Peak Annotation for Peaks**

The ‘annotatePeak’ function of ChIPSeeker v1.36.0 was utilized to annotate ATAC-seq peaks, using a range of ±3 kb to promoter-associated regions. Annotated peaks were assigned to the nearest gene based on the R-package TxDb.Hsapiens.UCSC.hg38.knownGene v3.17.0. DAR peaks were annotated in the same way.

**Hallmark Pathway Analysis with DAR**

The database of hallmark pathways for humans was downloaded from the R-package msigdbr v7.5.1. Hallmark pathway analysis was performed using fgsea v1.26.0 to investigate enriched pathways in the TAp36-OE group. The result was visualized via ggplot2 v3.4.3.

**TF Motif Analysis**

To identify potential TFs in the TAp63-OE group, we searched for TFs enriched at accessible sites using the ‘findMotifsGenme.pl’ function of HOMER v4.10.0 with the JASPAR 2020 database. We then visualized the top 10 de novo motifs for each group via ggplot2 v3.4.3.

**Cell lysate preparation for immunoblotting**

AsPC-1 cells were treated with MG132 (10 µM, Calbiochem), bortezomib (BTZ, 100 nM, Merck), E64d/Pep (10ng/ml, Sigma-Aldrich), Pepstatin A (10 ng/ml, Calbiochem), and Chloroquine diphosphate (CQ, 50 µM, Sigma-Aldrich) for 6 hrs, followed by a collection of cell lysates using M2 buffer (20 mM Tris-HCl (pH 7.5), 0.5% NP-40, 250 mM NaCl, 0.5 M EDTA, 0.25 M EGTA) with protease inhibitors cocktail. SNU410 cells were treated with recombinant human (rh)TNF-α (30 ng/ml, R&D system), recombinant mouse (rm)TNF-α (30 ng/ml, R&D system), rmIFN-α (200 ng/ml, R&D system), rhIFN-β (200 ng/ml, Peprotech), rhIFN-γ (200 ng/ml, Peprotech), and rhIL-1β (1 µg/ml, GenScript). Capan-1 cells were also treated with rhTNF-α (30 ng/ml, R&D system), rhIFN-β (200 ng/ml, Peprotech) and rhIFN-γ (200 ng/ml, Peprotech), and cell lysates were collected. After pre-treatment for 1 hour with Actinomycin D (1 µg/ml, Sigma-Aldrich) or Bay 11-7082 (20 µM Sigma-Aldrich), SNU410 or Aspc1 cells were treated with rhTNF-α (30 ng/ml, R&D system) for 15 min or 24 hrs, and followed by the collection of cell lysates for Immunoblotting.

**Immunoblotting**

The protein concentration of cell lysates was determined using Bradford assay (Bio-Rad, Hercules, CA, USA). Equal amounts of protein from each sample were resolved using sodium SDS-PAGE and transferred onto nitrocellulose membranes (EMD Millipore, Billerica, MA, USA). Immunoblots were blocked by incubation with 5% skim milk or bovine serum albumin in TBS-T buffer (25 mM Tris-HCl [pH 8.0], 150 mM NaCl, and 0.1% Tween 20) at room temperature for 1 h. The membranes were then incubated with anti-TP63 (∆Np63; Cell Signaling Technology, Danvers, MA, USA, Cat#: 39692), anti-TAp63 (Novus Biologicals, Littleton, CO, USA, Cat#: NBP3-11703), anti-TAp63-α/∆Np63-α (Cell Signaling Technology, Cat#: 13109), anti-∆Np63 (Cell Signaling Technology, Cat#: 67825), anti-NIK (Cell Signaling Technology), anti-HSP90 (Cell Signaling Technology), anti-LC3 II, (Sigma-Aldrich), anti-beta-Actin (Santa Cruz), anti-ZBP1 (Cell Signaling Technology), anti-VINCULIN (Sigma-Aldrich), IκBα (Cell Signaling Technology), and GAPDH (Novus Biologicals) on a shaker overnight at 4 °C. Protein was detected using an ECL detection kit (GE Healthcare, Piscataway, NJ, USA). The detailed information of the antibodies used to recognize TP63 isoforms are summarized as follows.

| Name | Vendor | Cat# | RRID | Specificity | Ref |
| --- | --- | --- | --- | --- | --- |
| TP63 | CST | 39692 | AB_2799159 | ○ TAp63 and ∆Np63 isoforms that contain exon 4, such as alpha, beta, and gamma | 12,13 |
| TAp63 | Novus Biologicals | NBP3-11703 | None | ○ Bind to TAp63 isoforms (alpha and gamma).  ○ This antibody does not recognize ∆Np63 isoforms. | None |
| TAp63-α / ∆Np63-α | CST | 13109 | AB_2637091 | ○ Bind to TAp63-α & ∆Np63-α  ○ This antibody does not recognize beta or gamma isoforms of TAp63 and ∆Np63 | 14,15 |
| ∆Np63 | CST | 67825 | AB_2799737 | ○ Bind to all ∆Np63 isoforms  ○ This antibody does not detect TAp63. | 16,17 |

**RNA sequencing**

The total RNA of mock-, TAp63-, and ΔNp63-transfected AsPC-1 and Panc-1 cells was isolated using an RNA isolation kit (Qiagen). RNA-seq libraries were prepared with 1 μg of total RNA from each sample using the Illumina TruSeq Stranded mRNA Sample Prep Kit (Illumina, San Diego, CA, USA). Indexed libraries were subjected to paired-end (2 × 100 bp) sequencing using Illumina NovaSeq (Illumina). All experiments were performed in duplicate. Raw sequencing data derived from mock, TAp63-OE, and ΔNp63-OE samples were processed using the AltAnalyze software.^18^ Transcript- and gene-level expression values (TPMs) and the summary data, including logFC values, generated by AltAnalyze, were used to compute the z-score. Expression z-scores of selected genes and transcripts were visualized as heatmaps using Morpheus software (https://software.broadinstitute.org/morpheus).

**Chromatin immunoprecipitation (ChIP) assay**

Chip assays were performed using Pierce Agarose ChIP Kit (Thermo Fisher Scientific, Massachusetts, USA) according to the manufacturer’s instructions. Briefly, SNU410 pancreatic cancer cells were treated with rhTNF-α (20 ng/ml, PeproTech) or control for 6 hr. After crosslinking, lysis, and DNA digestion process, immunoprecipitation was performed with anti-P50 (Cell Signaling Technology) anti-RelA (Cell Signaling Technology), and rabbit IgG (Cell Signaling Technology) control. Then, the purified DNA was isolated by washing, elution, and a DNA recovery process. Human TAp63 promoter regions from purified DNA were detected through qPCR. Primer sequences used were as follows: forward, CGGTTGGCTGAAAGGGAAAC, and reverse, ATGAGTGTCCACCCTGCTC.

**TAp63 reporter assay and co-culture system**

TAp63 promoter sequence (-685 to +125, GRCh38), which was reported to exhibit reporter activity by NF-κB,^12^ was inserted into pGL4.17 (luc2/neo, Promega). To establish TAp63 reporter cell, 293T cells in the 6 well plate (Corning) were co-transfected with TAp63-luc2/neo vector and thymidine kinase-renilla luciferase control vector using ViaFect (Promega), following the manufacturer’s instructions. To investigate the paracrine effect of inflammatory macrophage, THP-1 monocytes were differentiated using 150 nM of Phorbol 12-myristate 13-acetate (PMA, Sigma-Aldrich) for 1 day. Subsequently, the cells were polarized into inflammatory macrophages by incubation with 20 ng/ml of rhIFN-γ (PeproTech) and 100 ng/ml of LPS (Sigma-Aldrich) in the 6 transwell insert (membrane pore size: 0.4 µm, Corning) for 2 days. The inserts were transferred onto the top of the TAp63 reporter cell after treating the cells with IgG (50 ng/ml, Biolegend) or anti-TNFα (50 ng/ml, Biolegend), then the transwell was incubated for 1 day. After removal of the insert, luciferase activity was examined at 24 hrs using the dual-luciferase assay system (Promega).

**Statistical analysis**

Statistical tests were performed using GraphPad Prism 9 (GraphPad, La Jolla, CA, USA) or SPSS ver. 28.0 for Windows (SPSS Inc., Chicago, IL, USA). Statistical comparisons between two different groups were performed using the chi-square, Fisher’s exact, Mann-Whitney U tests, or Welch’s t-test. To compare multiple groups, statistical significance was assessed using analysis of variance (ANOVA), and adjustment for multiple comparisons was performed using the Kruskal-Wallis, Dunn’s multiple comparisons, or Sidak’s multiple comparisons tests, as deemed appropriate. Survival analyses for recurrence-free survival (RFS) and disease-specific survival (DSS) were performed using the KM method and log-rank test. Statistical significance was set at *p* < 0.05. All reported *p*-values were two-sided.

**References**

1. Lim SB, Tan SJ, Lim WT, Lim CT. Compendiums of cancer transcriptomes for machine learning applications. *Sci Data* 2019;6:194.

2. Bin Lim S, Chua MLK, Yeong JPS, Tan SJ, Lim WT, Lim CT. Pan-cancer analysis connects tumor matrisome to immune response. *NPJ Precis Oncol* 2019;3:15.

3. Bankhead A, 3rd, McMaster T, Wang Y, Boonstra PS, Palmbos PL. TP63 isoform expression is linked with distinct clinical outcomes in cancer. *EBioMedicine* 2020;51:102561.

4. Rashid NU, Peng XL, Jin C, et al. Purity Independent Subtyping of Tumors (PurIST), A Clinically Robust, Single-sample Classifier for Tumor Subtyping in Pancreatic Cancer. *Clin Cancer Res* 2020;26:82-92.

5. Subramanian A, Tamayo P, Mootha VK, et al. Gene set enrichment analysis: a knowledge-based approach for interpreting genome-wide expression profiles. *Proc Natl Acad Sci U S A* 2005;102:15545-15550.

6. Mootha VK, Lindgren CM, Eriksson KF, et al. PGC-1alpha-responsive genes involved in oxidative phosphorylation are coordinately downregulated in human diabetes. *Nat Genet* 2003;34:267-273.

7. Peng J, Sun BF, Chen CY, et al. Single-cell RNA-seq highlights intra-tumoral heterogeneity and malignant progression in pancreatic ductal adenocarcinoma. *Cell Res* 2019;29:725-738.

8. Hao Y, Hao S, Andersen-Nissen E, et al. Integrated analysis of multimodal single-cell data. *Cell* 2021;184:3573-3587 e3529.

9. Kim YB, Ahn JM, Bae WJ, Sung CO, Lee D. Functional loss of ARID1A is tightly associated with high PD-L1 expression in gastric cancer. *Int J Cancer* 2019;145:916-926.

10. Soares KC, Rucki AA, Wu AA, et al. PD-1/PD-L1 blockade together with vaccine therapy facilitates effector T-cell infiltration into pancreatic tumors. *J Immunother* 2015;38:1-11.

11. Chatterjee A, Upadhyay S, Chang X, Nagpal JK, Trink B, Sidransky D. U-box-type ubiquitin E4 ligase, UFD2a attenuates cisplatin mediated degradation of DeltaNp63alpha. *Cell Cycle* 2008;7:1231-1237.

12. Wu J, Bergholz J, Lu J, Sonenshein GE, Xiao ZX. TAp63 is a transcriptional target of NF-kappaB. *J Cell Biochem* 2010;109:702-710.

13. Yuan Y, Park J, Feng A, et al. YAP1/TAZ-TEAD transcriptional networks maintain skin homeostasis by regulating cell proliferation and limiting KLF4 activity. *Nat Commun* 2020;11:1472.

14. Luan Y, Yu SY, Abazarikia A, Dong R, Kim SY. TAp63 determines the fate of oocytes against DNA damage. *Sci Adv* 2022;8:eade1846.

15. Cappello A, Mancini M, Madonna S, et al. Extracellular serine empowers epidermal proliferation and psoriasis-like symptoms. *Sci Adv* 2022;8:eabm7902.

16. Lambert AW, Fiore C, Chutake Y, et al. DeltaNp63/p73 drive metastatic colonization by controlling a regenerative epithelial stem cell program in quasi-mesenchymal cancer stem cells. *Dev Cell* 2022;57:2714-2730 e2718.

17. Quintanal-Villalonga A, Taniguchi H, Zhan YA, et al. Comprehensive molecular characterization of lung tumors implicates AKT and MYC signaling in adenocarcinoma to squamous cell transdifferentiation. *J Hematol Oncol* 2021;14:170.

18. Emig D, Salomonis N, Baumbach J, Lengauer T, Conklin BR, Albrecht M. AltAnalyze and DomainGraph: analyzing and visualizing exon expression data. *Nucleic Acids Res* 2010;38:W755-762.
